# Probiotic *Bacillus subtilis* enhances silkworm (*Bombyx mori*) growth performance and silk production via modulating gut microbiota and amino acid metabolism

**DOI:** 10.1186/s42523-025-00473-1

**Published:** 2025-10-03

**Authors:** Chunjiu Ren, Yingchen Meng, Yangyang Liu, Yi Wang, Huizhen Wang, Yating Liu, Changjun Liu, Xin Fan, Shengxiang Zhang

**Affiliations:** 1 Department of Sericulture, College of Forestry, Shandong Agricultural University, Taian, 271018 China; 2 State Forestry and Grassland Administration Key Laboratory of Silviculture in Downstream Areas of the Yellow River, Shandong Agricultural University, Taian, 271018 China; 3 Department of Biotechnology and Engineering, College of Life Sciences, Shandong Agricultural University, Taian, 271018 China; 4 Department of Sericulture, Sericulture and Edible Fungus Institute, Yibin Academy of Agricultural Sciences, Yibin, 644699 Sichuan China

**Keywords:** Bacillus subtilis, Silkworm, Gut microbiota, Growth promotion, Amino acid metabolism, Artificial diet

## Abstract

**Background:**

Artificial diet-reared silkworms (*Bombyx mori*) exhibit reduced gut microbial diversity and impaired growth performance compared to mulberry-fed counterparts. While *Bacillus subtilis* is widely used as a probiotic in livestock and aquaculture, its impact on silkworms remains unexplored. This study investigates whether dietary supplementation with *B. subtilis* enhances larval development and elucidates the underlying mechanisms involving gut microbiota and metabolic pathways.

**Results:**

Supplementing artificial diets with *B. subtilis* (6 × 10^5^ CFU/g) significantly increased larval body weight by 9.1–22.1% during instar stages and improved feed utilization efficiency (FUE) by 4.09%–6.80% compared to controls. Cocoon quality metrics, including cocoon shell weight (+ 9.77% in females) and cocoon shell ratio (+ 6.56%), also improved. Mechanistically, *B. subtilis* did not colonize the midgut but transiently modulated gut physiology: it elevated midgut fluid pH and enhanced α-amylase, trypsin, and lipase activities. 16 S rRNA sequencing revealed reduced gut microbial diversity (Shannon index, *P* < 0.01) and shifts in community structure, with decreased abundances of potential pathogens (e.g., Pseudomonas) and commensals (e.g., Lactobacillus). Targeted metabolomics identified a 3.1-fold increase in phenylalanine levels in hemolymph, linked to upregulated aromatic amino acid metabolism pathways (KEGG). Dietary phenylalanine supplementation (0.4%) replicated *B. subtilis*-induced growth promotion, confirming its pivotal role in host-microbe interactions.

**Conclusions:**

*B. subtilis* enhances silkworm growth and silk production through multi-faceted mechanisms: reshaping gut microbiota composition, improving digestive enzyme activity, and elevating phenylalanine biosynthesis. These findings establish *B. subtilis* as a promising probiotic for optimizing artificial diet systems in Lepidoptera and highlight the central role of amino acid metabolism in insect-microbiome symbiosis.

**Supplementary Information:**

The online version contains supplementary material available at 10.1186/s42523-025-00473-1.

## Introduction

The silkworm (*Bombyx mori*) is an important economic insect, as it can produce silk, making a significant contribution to the development of the human textile industry and the enhancement of clothing standards. In addition, the silkworm serves as an important model organism for insects, particularly those belonging to the second largest insect order, Lepidoptera. It is widely used in scientific research, including genetics and molecular biology [1], developmental biology [2] and evolutionary studies [3], biotechnology and synthetic biology research [4], disease modeling and medical research [5, 6], and agricultural and ecological studies [7].

In recent years, with the continuous breakthroughs in cultivating silkworm varieties adaptable to artificial feed and the significant reduction in the cost of artificial feed for silkworms, the use of artificial feed for sericulture has rapidly developed. This has not only facilitated the rapid transformation and upgrading of the sericulture industry toward modern agriculture but also greatly promoted scientific research on silkworms as model organisms in the order *Lepidoptera*.

The insect gut harbors a substantial diversity of microorganisms, among which probiotic bacteria confer numerous beneficial biological effects on their host insects. These include facilitating food digestion [8], regulating host development [9, 10], influencing behavioral traits [11], and even participating in immune modulation [12, 13]. The multifaceted interactions between insects and probiotic bacteria are crucial for their survival and the maintenance of biodiversity.

Studies have demonstrated that under artificial diet rearing conditions, the bacterial diversity in the midgut of silkworms is significantly reduced, accompanied by a simplified microbial community structure [14–16]. This phenomenon may serve as a critical contributing factor to the observed declines in growth performance, developmental efficiency, and immune disease resistance in artificially diet-reared silkworms [15, 16]. Lactic acid bacteria (LAB) support the health of silkworm feed and mulberry, drive growth, and strengthen natural immunity to fight diseases [17]. Liang et al. [18] reported that the silkworm gut symbiont *Enterococcus casseliflavus* ECB140, which was isolated from the gut of silkworms reared on mulberry leaves, promoted host growth by facilitating the synthesis of L-tryptophan. In addition, supplementing the probiotic *Pediococcus pentosaceus* strain (ZZ61) in artificial feed for silkworm rearing can significantly increase the body weight and cocoon quality of silkworms [19]. These findings demonstrate that probiotic supplementation represents an effective strategy to increase the growth and development of the silkworm *Bombyx mori*. However, current research on the physiological effects of microorganisms on silkworms—particularly those reared on artificial diets—remains relatively limited.

*Bacillus subtilis* is a gram-positive bacterium with remarkable environmental adaptability that is ubiquitously distributed in diverse natural habitats, including food, soil, water, and animal digestive tracts [20]. *Bacillus subtilis is generally considered nonpathogenic to animals and* can secrete various digestive enzymes and antimicrobial compounds [21]. It has been extensively utilized as a microbial additive in the fields of human gastrointestinal health [22, 23], livestock, poultry, and aquaculture [24–26] and as a biological control agent for plant protection [27, 28]. Although *Bacillus subtilis* is well established as a broad-spectrum probiotic, its potential growth-promoting effects on silkworms (*Bombyx mori*) reared on artificial diets remain unexplored.

In this study, we demonstrated that dietary supplementation with *Bacillus subtilis* significantly increased larval body weight and cocoon yield in *Bombyx mori*. Furthermore, multiomics analyses revealed the underlying mechanisms of its probiotic effects. Investigating their interactions would not only enhance the efficiency and economic viability of artificial diet-based sericulture but also contribute to a deeper understanding of bacterial-insect ecological relationships.

## Materials and methods

### Bacterial strains and culture conditions

The *Bacillus subtilis* strains used in this study were obtained from Baolai-Leelai Biological Engineering Co., Ltd. (Taian, China). The 16 S rDNA sequence of the bacteria was sequenced and subsequently aligned with the NCBI GenBank database, and an evolutionary tree was constructed via the software MEGA-10. The bacteria were cultured in LB medium for 18 h at 37 °C and centrifuged (5000 rpm, 5 min) to obtain the precipitate. The precipitate was subsequently washed twice with normal saline and suspended at a concentration of 10^8^ CFU/mL for further use.

### Colonization identification of Bacillus subtilis

With the use of GFP-expressing *Bacillus subtilis* (*BS*) for colonization assessment, the preparation method for GFP-expressing *BS* is detailed below [29]. The activated *BS* was transferred to a medium (containing 50 mL of LB medium and 0.5 M sorbitol) and cultured at 37 °C and 200 rpm for 12 h. Then, the culture was incubated on ice for 10 min, followed by centrifugation at 4 °C and 5,000 rpm for 8 min. The centrifuged pelleting mixture was resuspended in 30 mL of precooled electroporation medium (ETM, containing 0.5 M sorbitol, 0.5 M mannitol and 10% glycerol), followed by repeated centrifugation under identical conditions (5,000 rpm, 4 °C, and 8 min), and the supernatant was discarded 3 times. The final pellet (competent *BS*) was resuspended in 0.5 mL ETM and stored at -80 °C. Then, 60 µL of competent *BS* was thawed on ice and immediately supplemented with 50 ng of the GFP expression vector (pHAPII-GFP). The mixture was incubated on ice for 5 min prior to transfer into a precooled 1 mm gap electroporation cuvette. A single electrical pulse (2.0 kV, 25 µF, 200 Ω) was applied. Following electroporation, 1 mL of RM medium (preequilibrated to 37 °C) was added, and the cells were cultured at 37 °C and 200 rpm for 3 h. Transformants were selectively plated on ampicillin-containing LB medium. Aseptically collected *BS* strains exhibiting stable green fluorescence were orally administered to silkworms. After a 6-hour period of starvation, the midgut intestinal fluid was examined with a fluorescence microscope (Nikon-Ni U, Japan) to detect the presence of GFP-derived fluorescence.

### Silkworm rearing and grouping

The silkworm (strain Guangshi No. 1) larvae were reared all instars via an artificial diet (mulberry leaf powder 30%, corn meal 27%, soybean meal 35%, vitamins 3%, inorganic salts 3%, and preservatives 1%) [30] under standard conditions (27 ± 1 °C and a relative humidity of 75–80%).

Five groups were set up: CK (treated with no *Bacillus subtilis*), BS-1 (treated with *Bacillus subtilis* at a concentration of 6 × 10^4^ CFU/g diet), BS-2 (treated with 6 × 10^5^ CFU/g *Bacillus subtilis*), BS-3 (treated with 6 × 10^6^ CFU/g *Bacillus subtilis*) and BS-4 (treated with 6 × 10^7^ CFU/g *Bacillus subtilis*). The bacteria were added at the beginning of the 4th instar of the silkworm, and the diet containing *Bacillus subtilis* was replaced with a common artificially extruded pellet diet at the beginning of the 5th instar.

### Determination of body weight, feed utilization efficiency (FUE) and cocoon quality

The body weights of the larvae were measured at day 0 of the 4th instar (0D4I), day 4 of the 4th instar (4D4I), day 3 of the 5th instar (3D5I), and day 6 of the 5th instar (6D5I). The FUE (FUE = body weight gain/feed intake×100%) was determined during the 4th instar. The cocoon weight, cocoon shell weight and cocoon shell ratio (cocoon shell ratio = cocoon shell weight/cocoon weight× 100%) were measured on the 7th day after cocooning. Each group was set with 3 replicates, with 50 silkworms, including 25 males and 25 females, in each repetition.

### Determination of the pH and intestinal digestive enzyme activity of the digestive juices

Ten silkworms were randomly selected from each group on day 4 of the 4th instar and day 3 of the 5th instar, and their intestinal digestive juices were obtained on ice. Three volumes of ice-cold PBS were added to the digestive juices, which were subsequently centrifuged at 3500 rpm for 5 min. The supernatant was then used to determine the pH of the digestive juices and the activity of the digestive enzymes. Three replicates were conducted for each treatment. The activities of α-amylase, lipase and trypsin were measured with appropriate commercial reagent kits (Nanjing Jiancheng Biotechnic Institute, Nanjing, China).

### Analysis of the intestinal microbial composition

Ten silkworms were randomly selected from the CK group and BS-2 group at day 4 of the 4th instar and day 3 of the 5th instar, and the midgut tissue was dissected directly into a 2-mL sterile EP tube after the surface had been disinfected with 75% ethanol. The entire process was carried out on ice. Total DNA was extracted via an Omega Mag-bind soil DNA kit (Omega Bio-Tek, Norcross, GA, USA).

The PCR system contained 0.25 µL of DNA polymerase (Vazyme, Nanjing, China), 5 µL of 5× reaction buffer, 5 µL of 5 × Q5@ High GC Enhancer, 2 µL of dNTPs (10 mM), 10 ng of DNA template, and 1 µL of each primer (5 mM) and was subsequently carried out on a Biometra 070‒851 PCR instrument (Biometra, Göttingen, Germany). Notably, the 16S rRNA primers for midgut bacteria were 341F (5’- CCTACGGGNGGCWGCAG-3’) [31] and 806R (5’- GGACTACHVGGGTATCTAAT-3’), whereas those for fecal bacteria were 799 F (5’- AACMGGATTAGATACCCKG-3’) and 1193R (5’- ACGTCATCCCCACCTTCC-3’) [32]. The PCR procedure was carried out under the following conditions: an initial denaturation step at 95 °C for 5 min, followed by 30 cycles of 95 °C for 1 min, 60 °C for 1 min, and 72 °C for 1 min, and a final hold at 72 °C for 7 min.

The quality of the amplification product was assessed via 2% agarose gel electrophoresis, and the PCR product was purified with AMPure XP Beads (Beckman, CA, USA) and quantified via Qubit 3.0. The sequencing library was constructed via the Illumina DNA Prep Kit (Illumina, CA, USA), and qualified libraries were sequenced at Gene Denovo Bio-Technology Co., Ltd. (Guangzhou, China) via an Illumina NovaSeq 6000 sequencer (Illumina, San Diego, USA). The raw data were uploaded to the NCBI Sequence Read Archive (SRA) database (accession numbers: PRJNA1248733 and PRJNA1247772).

The raw reads were quality filtered via FASTP (version 0.18.0) to obtain clean reads, and FLASH (version 1.2.11) was used to assemble the clean reads into tags [33]. Then, high-quality clean tags were obtained through denoising, and UPARSE (version 9.2.64) was used to cluster the clean tags into operational taxonomic units (OTUs) on the basis of a similarity threshold of ≥ 97% [34]. The tag sequence with the highest abundance was selected as the representative sequence for each OTU. Bioinformatics analysis of the gut microbiota was conducted via the OmicsMart online platform (https://www.omicsmart.com).

### Targeted metabolic analysis of amino acids

Hemolymph samples from silkworms in the CK group and BS-2 group were collected at day 4 of the 4th instar. One hundred microlitres of each hemolymph sample was mixed with 900 µL of 80% methanol (containing tryptophan-d5 as an internal standard), vortexed for 3 min, and then centrifuged at 12,000 rpm for 10 min at 4 °C. The supernatant was filtered through a 0.22 μm filter membrane and diluted 50 times with 80% methanol before on-machine detection. The metabolite composition of the prepared samples was analyzed via an AB SCIEX 5500 QQQ UPLC‒MS liquid chromatography‒mass spectrometry instrument (SCIEX, USA), followed by bioinformatics analysis at Gene Denovo Bio-Technology Co., Ltd. (Guangzhou, China). The raw data were uploaded to the NGDC (National Genomics Data Center) OMIX (Open Archive for Miscellaneous Data) database (accession number: OMIX009822).

### Effects of phenylalanine supplementation on silkworm body weight and cocoon quality

The silkworm (strain Guangshi No. 1) larvae were reared on an artificial extruded pellet diet as described in Sect. “Silkworm rearing and grouping”. Phenylalanine was supplemented at the beginning of the 5th instar of the silkworm, with dietary concentrations set at 0.2%, 0.4%, and 0.8% relative to the dry weight of the artificial diet matrix. The body weights of the larvae were measured at day 1–5 of the 5th instar, and the cocoon weight, cocoon shell weight and cocoon shell ratio were measured at day 7 after cocooning. Sixty female silkworms were set in each group, with three repetitions set up in each.

### Statistical analysis

The data were analyzed by one-way analysis of variance (ANOVA), followed by the Student–Newman–Keuls stepwise multiple comparisons test to detect significant differences via SPSS 20.0 (SPSS Inc., Chicago, IL, USA) and are expressed as the mean ± standard error of the mean. Differences between means were considered significant when *P* < 0.05.

## Results

### Identification of Bacillus subtilis strains

16 S rDNA sequencing and evolutionary analysis revealed that the *Bacillus subtilis* strain used in the present study (named *BS*) and *Bacillus subtilis* MN689681 confirmed that the *BS* strain was the *Bacillus subtilis* MN689681 strain.

**Fig. 1:**
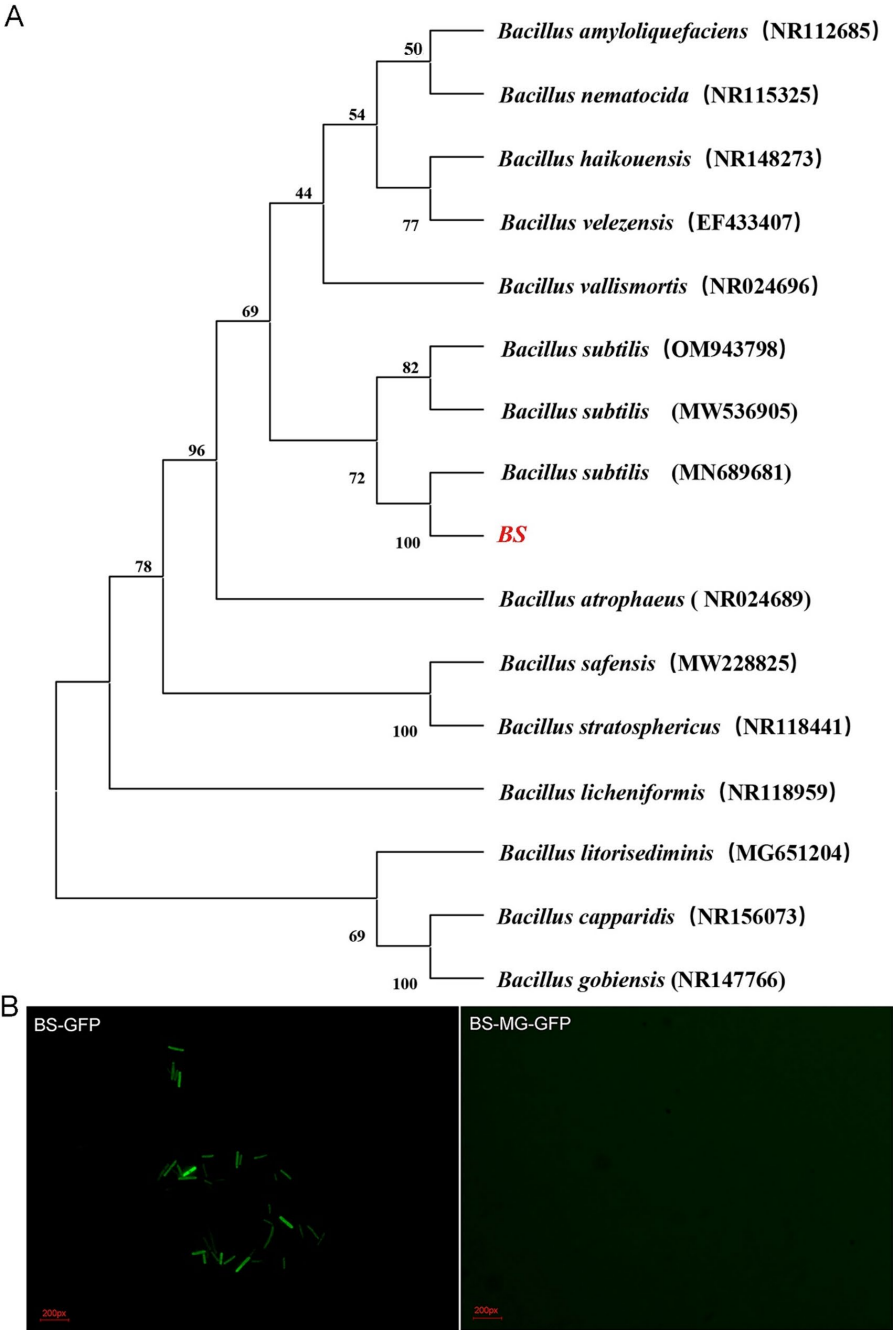
Evolutionary analysis and colonization identification of *Bacillus subtilis* strains. (**A**) The 16 s rDNA gene-based phylogenetic tree (BS, abbreviation of *Bacillus subtilis* strain used in this study). (**B**) Identification of colonization of *Bacillus subtilis* based on GFP in the midgut of silkworm. (BS-GFP, the GFP fluorescence of *Bacillus subtilis* cultured in vitro; BS-MG-GFP, the GFP fluorescence of *Bacillus subtilis* in the midgut of silkworm)

As shown in Fig. 1B, *BS* exhibited green fluorescence under a fluorescence microscope, indicating the successful transfer of the GFP vector into the bacteria. When silkworms were fed *BS* carrying the GFP vector, no green fluorescence was detected in their midgut fluid six hours later, implying that *BS* was unable to colonize the midgut of silkworms.

### Bacillus subtilis promotes the growth of silkworms and improves cocoon quality

**Fig. 2:**
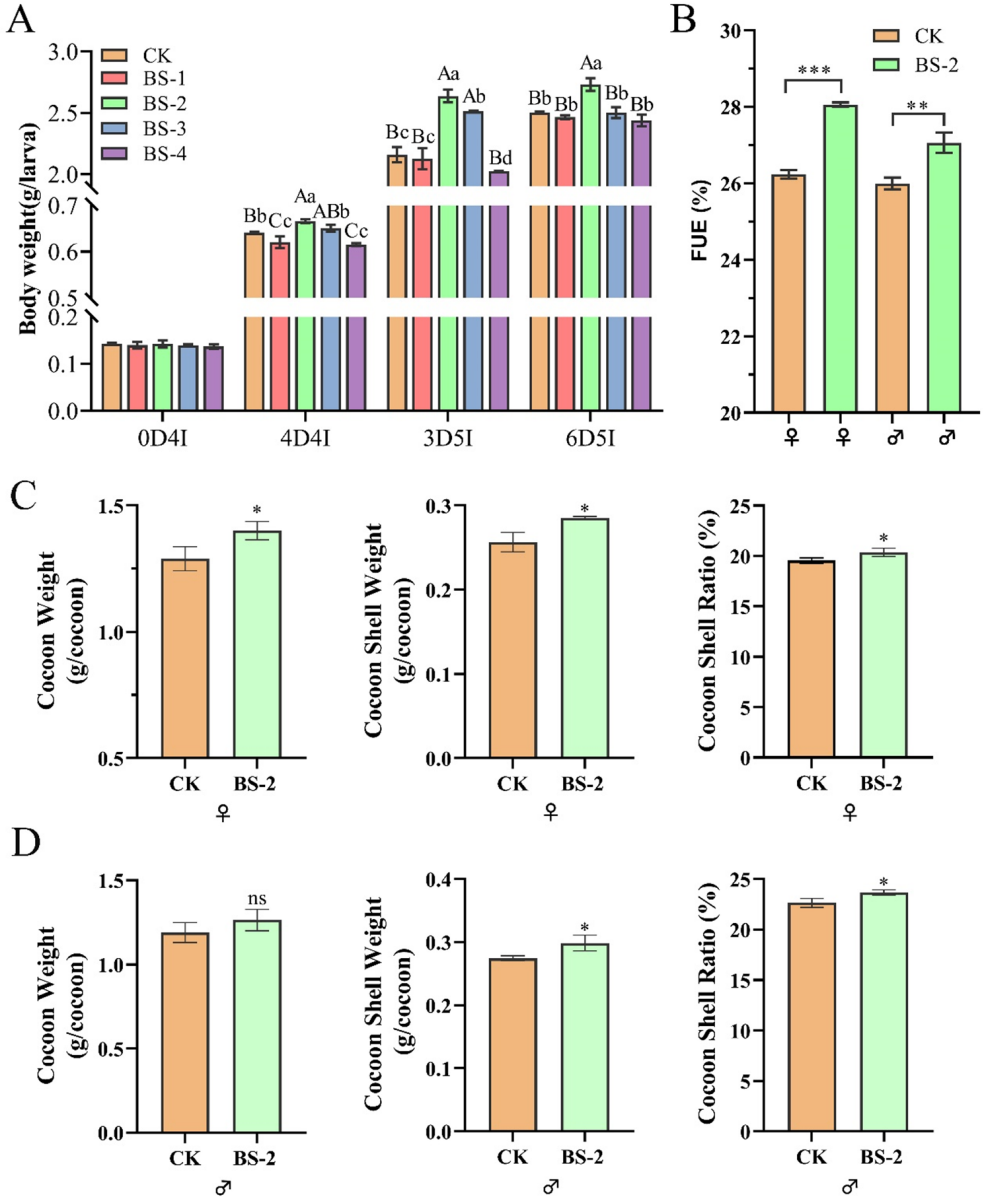
Effect of *Bacillus subtilis* supplementation on the body weight and FUE of silkworm at the 4th instar. (**A**) The larval body weight at day 0 of 4th instar, day 4 of 4th instar, day 3 of 5th instar and day 6 of 5th instar. (**B**) The FUE at day 4 of the 4th instar. (**C**) The cocoon quality of female silkworms at day 7 after cocooning. (**D**) The cocoon quality of male silkworms at day 7 after cocooning. A one-way ANOVA was used to analyze the significance of differences, and results are expressed as mean ± SEM. *P < 0.05, **P < 0.01, ***P < 0.001. Capital letters denote significant differences at *P* < 0.01. Lowercase letters denote significant differences at *P* < 0.05. ns, no significant difference

The body weights of silkworms in the BS-2 group were significantly greater than those in the other groups at day 4 of the 4th instar (4D4I) (+ 3.90%), day 3 of the 5th instar (3D5I) (+ 22.12%) and day 6 of the 5th instar (6D5I) (+ 9.10%) (*P* < 0.01) (Fig. 2A). There was no significant difference in the cocoon weight of male silkworms between the BS-2 group and the CK group (*P* > 0.05), but the cocoon shell weight and cocoon shell ratio were significantly greater than those of the CK group (+ 7.23% and + 4.52%, respectively) (*P* < 0.05), whereas the cocoon weight, cocoon shell weight, and cocoon shell ratio of female silkworms in the BS-2 group were significantly greater than those of the CK group (+ 8.53%, + 9.77% and + 6.56%, respectively) (Fig. 2C, D). Moreover, the FUE (Fig. 2B) of both female and male silkworms in the BS-2 group was significantly greater (+ 6.80% and + 4.09%, respectively) than that in the CK group at the 4th instar (*P* < 0.001, *P* < 0.001).

### Effects of Bacillus subtilis supplementation on the pH and intestinal digestive enzymes in the midgut intestinal fluid of silkworms

**Fig. 3:**
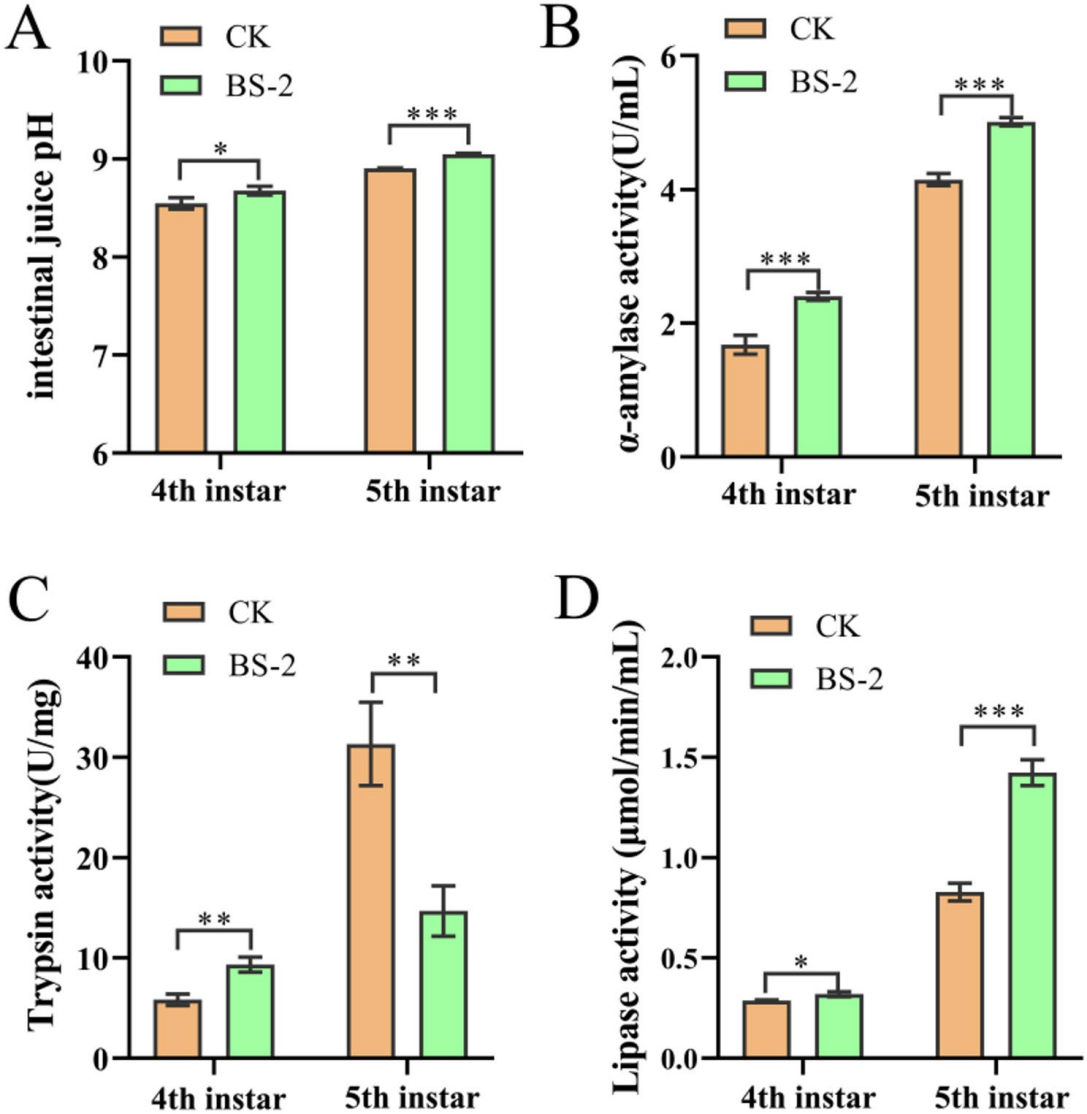
Effect of *Bacillus subtilis* supplementation on the pH and intestinal digestive enzymes of silkworm intestinal fluid. (**A**) The intestinal juice pH of midgut at day 4 of 4th instar and day 3 of 5th instar (*n* = 3, each sample contains 10 larva). (**B**-**D**) The gut digestive enzyme activity of α-amylase, trypsin and lipase at day 4 of 4th instar and day 3 of 5th instar (*n* = 3, each sample contains 10 larva). A one-way ANOVA was used to analyze the significance of differences, and results are expressed as mean ± SEM. *P < 0.05, **P < 0.01, ***P < 0.001

As shown in Fig. 3A, the pH of the intestinal fluid of the BS-2 group at both the 4th instar and 5th instar stages was significantly greater than that of the CK group (*P* < 0.05 and *P* < 0.001, respectively). The intestinal digestive enzyme activity was also measured (Fig. 3B-D). The α-amylase, trypsin and lipase activities were 43.10% (*P* = 0. 001), 60.00% (*P* = 0. 003), and 11.51% (*P* = 0. 012), respectively, were greater in the BS-2 group than in the CK group at the 4th instar period, while the α-amylase and lipase activities were 20.86% (*P* < 0.001), and 71.88% (*P* < 0.001) were also greater at the 5th instar period. However, the trypsin enzyme activity was 53.19% (*P* = 0. 004) was lower in the BS-2 group than in the CK group at the 5th instar.

### Effects of Bacillus subtilis supplementation on microbial structure and composition in the midgut of silkworms

**Fig. 4:**
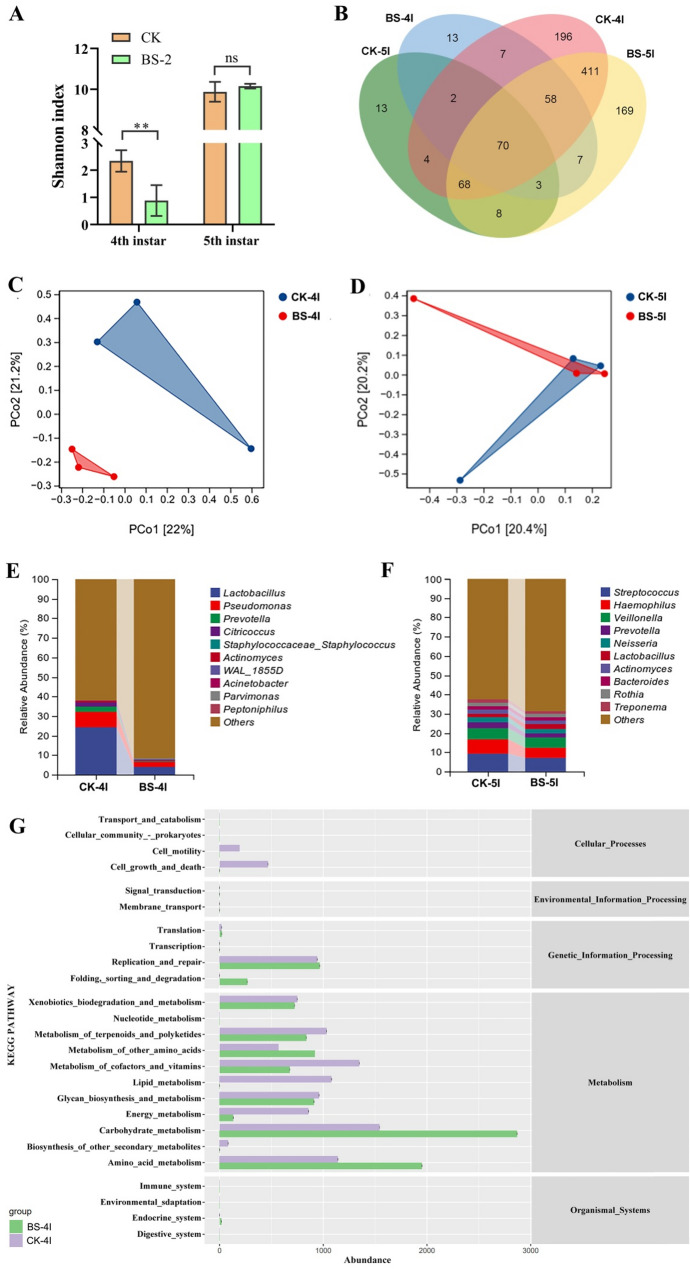
Effect of *Bacillus subtilis* supplementation on microbial structure and composition in the midgut of silkworms. (**A**) Shannon index for community diversity the CK and BS-2 groups (**P < 0.01; ns, no significant difference.). (**B**) The Venn diagram between the CK (CK-4I, CK group at day 4 of 4th instar; CK-5I, CK group at day 3 of 5th instar) and BS-2 (BS-4I, BS-2 group at day 4 of 4th instar; BS-5I, BS-2 group at day 3 of 5th instar) groups. (**C**) PCA of differences in gut microbiota of larva between the CK-4I and BS-4I groups. (**D**) PCA of differences in gut microbiota of larva between the CK-5I and BS-5I groups. (**E**) The relative bacterial abundance at genus-level in the midgut of the silkworms between the CK-4I and BS-4I groups. (**F**) The relative bacterial abundance at genus-level in the midgut of the silkworms between the CK-5I and BS-5I groups. (**G**) KEGG analysis of gut bacterial metabolic pathways of the silkworms between the CK-4I and BS-4I groups

The species diversity of the intestinal bacteria was calculated with several indices [e.g., the Shannon, Simpson and Chao1 indices (Table S1), and the Shannon index was visualized (Fig. 4-A)]. The community diversity of the BS-2 group was significantly lower than that of the CK group (*P* < 0.01) during the 4th instar period. However, after *Bacillus subtilis* supplementation was stopped at the 5th instar stage, there was no significant difference in species diversity between the BS-2 group and the CK group.

A Venn diagram shows the distribution of bacterial genera between the two groups (Fig. 4B). At the 4th instar stage, there were 93 unique bacteria in the CK group and 85 unique bacteria in the BS-2 group, with a total of 77 bacteria shared between the two groups. However, at the 5th instar stage, there were 209 unique bacteria in the CK group and 187 unique bacteria in the BS-2 group, with a total of 1003 bacteria shared between the two groups. This finding indicates that the number of bacteria at the genus level in the CK group notably outnumbered that in the BS-2 group and that supplementation with *Bacillus subtilis* reduces the diversity of gut microorganisms.

At the phylum level, in both the 4th instar and 5th instar groups, *Firmicutes* was the most abundant phylum, followed by *Proteobacteria* and *Actinobacteria*, but the proportions were slightly different between the groups (Fig. S1). At the genus level (Fig. 4E), the top 3 dominant genera in the CK group at the 4th instar stage were *Lactobacillus* (24.0%), *Pseudomonas* (8.2%) and *Citricoccus* (2.5%). However, the top 3 dominant genera in the BS-2 group at the 4th instar stage were *Lactobacillus* (3.9%), *Pseudomonas* (2.8%) and *Prevotella* (0.1%). The abundance of *Lactobacillus* and *Pseudomonas* in the treatment group was significantly lower than that in the control group (3.92% vs. 24.0%, *P* < 0.05; 2.8% vs. 8.2%, *P* < 0.05). At the 5th instar, the top 3 dominant genera in both the CK group and BS-2 group were *Streptococcus* (9.3% vs. 6.9%), *Haemophilus* (7.3% vs. 5.6%) and *Veillonella* (5.7% vs. 5.0%), and the differences between the two groups were not significant (Fig. 4F).

Furthermore, intestinal bacterial function prediction was conducted via PICRUSt2, and a deeper understanding of the relationships between the gut microbiota of silkworms and their growth and development was obtained. The results revealed that the metabolic function of silkworm intestinal bacteria significantly changed after feeding with *Bacillus subtilis* during the 4th-instar period. Upon further analysis of the KEGG secondary metabolic pathways whose expression significantly differed during the 4th instar period in silkworms, carbohydrate metabolism, amino acid metabolism and metabolism of other amino acids were upregulated (Fig. 4G). These intestinal bacterial functions are crucial for promoting the growth and development of silkworms. Moreover, there was a similar difference in the function of the intestinal flora between the two groups of silkworms after they stopped feeding with *Bacillus subtilis* at the 5th instar (Fig. S2).

### Effects of Bacillus subtilis supplementation on amino acid metabolism in silkworms

**Fig. 5:**
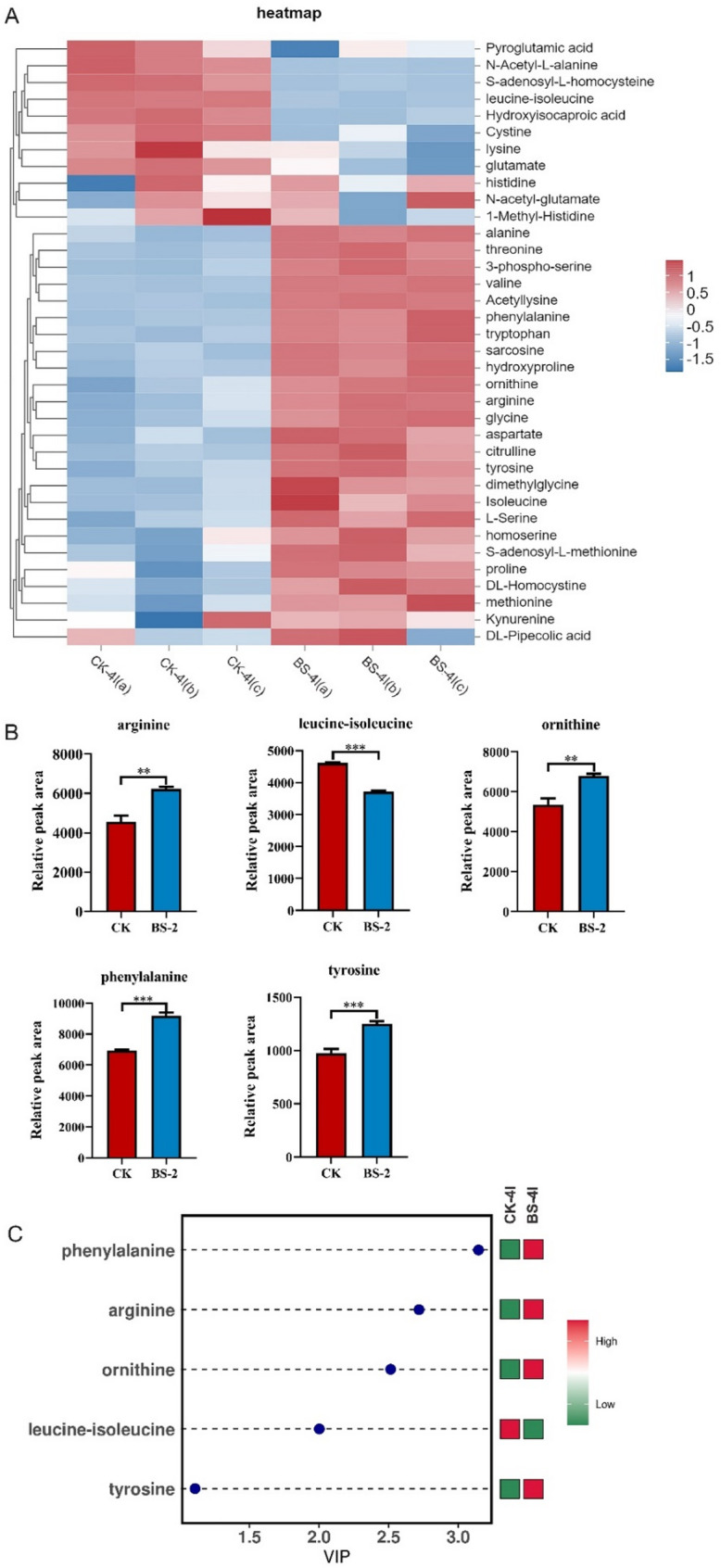
Targeted metabolomics analysis of amino acids in the hemolymph of silkworm. (**A**) Heatmaps display of the relative abundances of 36 kinds of amino acids between CK group and BS-2 group. (**B**) Relative abundances of the 5 amino acids showing significant differences between CK group and BS-2 group. (**C**) The significantly different amino acids to the variable importance in projection (VIP) score of OPLS-DA at VIP > 1.0 and a *P*-value < 0.05. Results are expressed as mean ± SEM. *P < 0.05, **P < 0.01, ***P < 0.001

Among the 36 amino acids and their metabolites identified through targeted metabolomics (Fig. 5A), five exhibited significant differences in their relative contents in the blood of silkworm larvae in the treated group compared with those in the control group (Fig. S3). Specifically, tyrosine, ornithine, arginine, and phenylalanine were significantly upregulated (*P* < 0.001, *P* < 0.05, *P* < 0.01, *P* < 0.001, respectively), whereas leucine-isoleucine was significantly downregulated (*P* < 0.001) (Fig. 5B).

Moreover, we analyzed the VIP (variable important in projection) of differential amino acids. The higher the VIP value is, the greater the importance of the differential amino acid. As illustrated in Fig. 5C, the VIP values of the five distinct amino acids are all greater than 1 (VIP > 1), with phenylalanine exhibiting the highest VIP value (VIP > 3). It was speculated that phenylalanine may play a significant role in promoting the growth of silkworms through supplementation with *Bacillus subtilis*.

### Effects of phenylalanine supplementation on silkworm body weight and cocoon quality

The effects of phenylalanine supplementation on larval development and cocoon quality in *Bombyx mori* were investigated through dose‒dependent trials, with the aim of biochemically validating amino acid-targeted metabolomics findings and elucidating amino acid-mediated growth regulation. The results demonstrated that all three dietary concentrations of phenylalanine (0.2%, 0.4%, and 0.8% w/w) significantly increased larval body weight (*P* < 0.01). Furthermore, compared with the control diet, phenylalanine supplementation markedly improved whole cocoon weight (+ 15.03–20.05%) and cocoon shell weight (+ 17.91–21.75%). These findings, which are similar to those obtained from *Bacillus subtilis* supplementation, not only corroborate the targeted metabolomics data but also establish that single-component phenylalanine fortification substantially optimizes rearing performance in artificial diet-based sericulture systems.

**Fig. 6:**
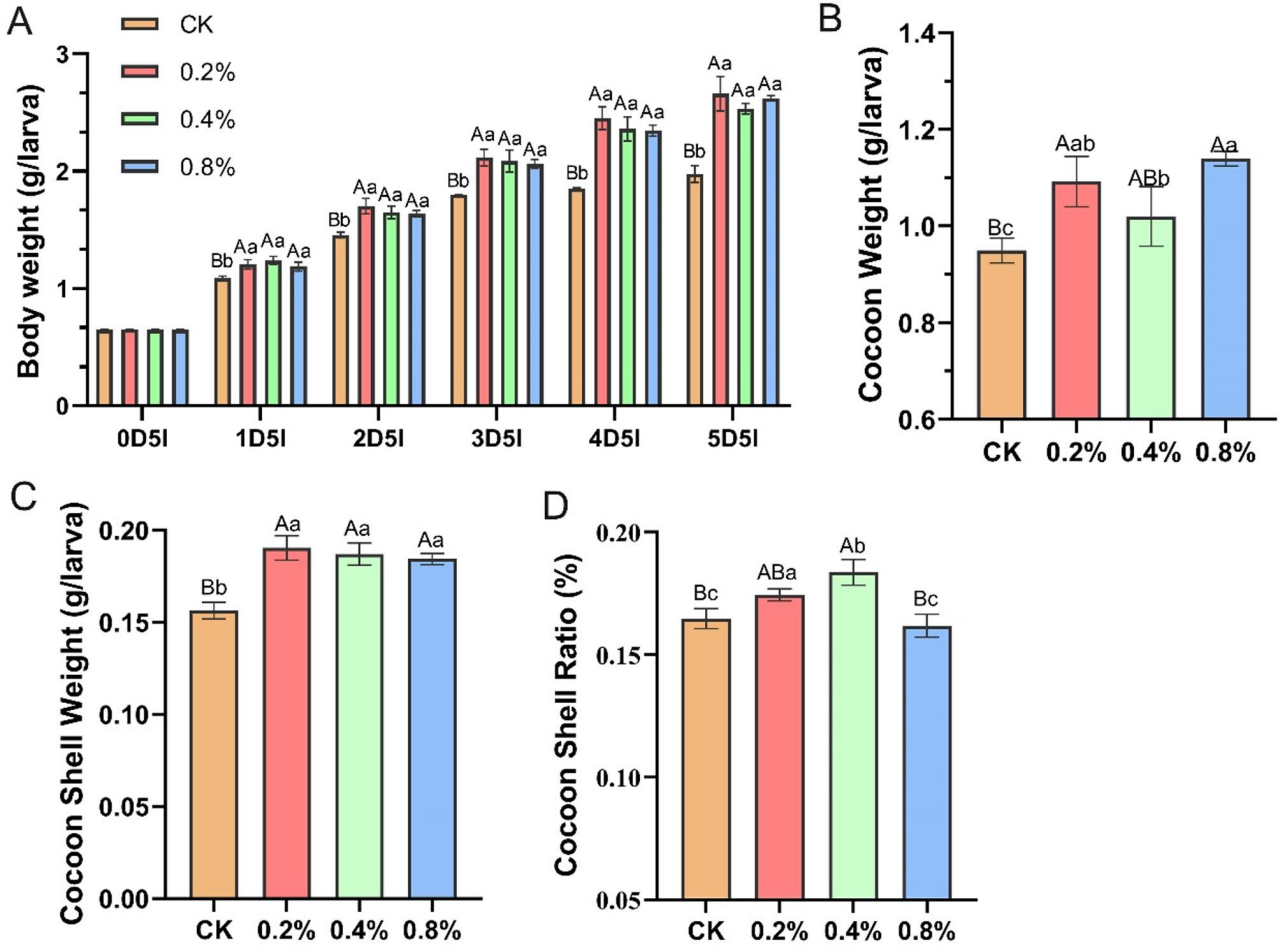
Supplementation of phenylalanine improves the body weight and cocoon quality of silkworms. (**A**) The larval body weight at 0–5 day of 5th instar. (**B**) The cocoon weight of female silkworms at day 7 after cocooning. (**C**) The cocoon shell weight of female silkworms at day 7 after cocooning. (**D**) The cocoon shell ratio of female silkworms at day 7 after cocooning. A one-way ANOVA was used to analyze the significance of differences, and results are expressed as mean ± SEM (*n* = 3, 60 female silkworms were set in each group). Capital letters denote significant differences at *P* < 0.01. Lowercase letters denote significant differences at *P* < 0.05

## Discussion

*Bacillus subtilis* is a commonly used microbial feed additive in the livestock industry that effectively regulates the animal gut microbiota, enhances immune function, and improves metabolism. In this study, we found that dietary supplementation with *Bacillus subtilis* also improved the gut microbiota of silkworms reared on artificial diets and promoted their growth and development. Additionally, we observed several unique characteristics that were different from those reported in previous studies.

Unlike most probiotics, which function through intestinal colonization, *Bacillus subtilis* could no longer be detected for intestinal colonization as early as 6 h after feeding (Fig. 1B). Recent studies have shown that even without colonization, *Bacillus subtilis* can still achieve probiotic functions through mechanisms such as metabolites, immune regulation, and transient gut microbiota remodeling [35–37]. *Bacillus subtilis* can secrete various digestive enzymes [38–40] (e.g., amylases and proteases), degrade antinutritional factors, and improve feed utilization [41]. In this study, we found that after feeding with *Bacillus subtilis*, the activities of α-amylase, trypsin, and lipase in the silkworm intestine were significantly increased. These substances can improve the digestive capacity of the organism for feed, thereby increasing feed efficiency (Fig. 2B). Additionally, this study revealed that feeding with *Bacillus subtilis* increased the pH of the midgut fluid of silkworms. Since the silkworm intestine itself is an alkaline environment, an appropriate increase in pH may increase the digestive capacity [42], which could also be one of the reasons for the probiotic effects of *B. subtilis*.

Reconstructing the structure of the gut microbiota community may be another important reason for the probiotic effect of *B. subtilis*. The gut microbiota diversity index revealed that the Shannon index of the *Bacillus subtilis* treatment group was significantly lower than that of the control group at the 4th instar (Fig. 4A), suggesting that this bacterium may alter the microbiota community structure through competitive exclusion [37] or by secreting antibacterial substances [43, 44]. Venn diagram analysis revealed that the number of unique bacterial genera in the treatment group (85) was less than that in the control group (93), while the proportion of shared genera reached 77%, further indicating that the addition of *Bacillus subtilis* reduced the diversity of the silkworm gut microbiota (Fig. 4B). Previous studies have shown that the dominant phyla in silkworm life stages include *Proteobacteria*, *Firmicutes*, *Actinobacteria*, and *Bacteroidetes* [45–46]. In this study, the dominant phyla in the midgut of silkworms in both treatment groups were also *Proteobacteria*, *Firmicutes*, *Actinobacteria*, and *Bacteroidetes*, which is consistent with the findings of Chen et al. [46]. At the genus level, the addition of *Bacillus subtilis* reduced the abundance of *Lactobacillus* and *Pseudomonas*. Many bacteria of the *Pseudomonas* genus, which is an important genus within the *Proteobacteria*, are known to be pathogens or opportunistic pathogens, often causing diseases when the host’s immune system is compromised [47, 48]. Therefore, a reduction in *Pseudomonas* abundance decreases the risk of silkworms contracting disease and promotes their growth to some extent. *Lactobacillus*, a representative genus of *Firmicutes*, plays a crucial role in promoting digestion and metabolism [49] and regulating immunity [50] and is commonly found in silkworm midguts. Surprisingly, the abundance of *Lactobacillus* in the midgut of silkworms in the treatment group was significantly lower than that reported in previous studies [19], which may be related to an increase in the pH of the gut fluid [51–52]. Notably, the gut microbiota diversity recovered to control levels by the 5th instar (Fig. 4A), suggesting that the regulatory effect of *B. subtilis* is time limited, possibly due to the removal of probiotics from the diet and the self-regulatory capabilities of the host microbiome. Additionally, the growth-promoting effect of *Bacillus subtilis* addition may not be limited to changes in the abundance of one or a few genera but rather may result from alterations in the entire microbial community system.

KEGG pathway enrichment analysis revealed that dietary supplementation with *Bacillus subtilis* significantly upregulated pathways related to carbohydrate metabolism, amino acid metabolism, and lipid metabolism in silkworms (Fig. 4G). Notably, the phenylalanine metabolism pathway was particularly strengthened—targeted metabolomics revealed that phenylalanine levels in the treatment group were 3.1 times higher than those in the control group (VIP > 3, *P* < 0.001; Fig. 5C). As an aromatic amino acid, phenylalanine serves not only as an essential precursor for protein synthesis but also as a precursor for neurotransmitters such as dopamine. It plays complex and diverse roles in insect physiological metabolism [53], behavioral regulation [54, 55], stress resistance and defense [56], and evolutionary adaptation [57]. Studies have shown that the absence of endosymbiotic bacteria in *Blattella germanica* significantly alters the metabolic pathways of phenylalanine and tyrosine [58]. In *Spodoptera frugiperda*, abnormal phenylalanine metabolism may inhibit the tricarboxylic acid (TCA) cycle, reducing GTP production and causing an energy supply imbalance in larvae [59]. In this study, *Bacillus subtilis* may regulate silkworm amino acid metabolism and energy metabolism by promoting phenylalanine synthesis, thereby promoting silkworm growth. Studies have indicated that, in comparison to mulberry leaves, the amino acid content in artificial diets for silkworms is unbalanced. By solely supplementing with specific amino acids, such as glycine, the breeding performance of silkworms can be notably enhanced [60]. In this research, the solitary addition of phenylalanine was found to significantly boost the growth and development of silkworms, achieving effects akin to those observed with Bacillus subtilis. This finding not only validates the pivotal role of phenylalanine in the probiotic action of *Bacillus subtilis* on silkworms but also suggests that the phenylalanine content in artificial feed for silkworms may likewise be unbalanced.

Furthermore, targeted metabolic data further suggested that following the supplementation of *Bacillus subtilis*, the concentrations of tyrosine, arginine, and ornithine in the hemolymph of silkworms have also been elevated. Studies have demonstrated that tyrosine is the hydroxylated derivative of phenylalanine [61], and an elevated phenylalanine concentration indirectly facilitates an increase in tyrosine concentration. Together, they synergistically enhance the growth and development of silkworms [62]. Arginine plays a pivotal role in the nitrogen metabolism cycle of silkworms, being essential for the efficient utilization of nitrogen resources in silk glands to synthesize silk proteins [63]. Ornithine, meanwhile, serves as the immediate precursor for the synthesis of polyamines [64]. Collectively, these two amino acids form the ornithine-ammonia cycle within silkworm metabolism, exerting a direct impact on growth and development by facilitating nitrogen resource allocation, silk protein synthesis, and polyamine generation [64]. Leucine and isoleucine serve as crucial regulatory factors in the synthesis silk protein. The reduction in their levels within the hemolymph of silkworms suggests a more efficient silk protein synthesis process [65]. Consequently, we postulate that the augmented metabolism of tyrosine, arginine, and ornithine, induced by the administration of *Bacillus subtilis*, could potentially constitute another significant factor contributing to enhanced silkworm growth and development.

## Conclusion

This study systematically reveals the biological effects of *Bacillus subtilis* in promoting the growth and development of silkworms reared on artificial feed through a multidimensional regulatory mechanism. Research has revealed that although *Bacillus subtilis* fails to colonize the host intestine, it can significantly increase larval weight, feed utilization efficiency, and cocoon silk quality by regulating digestive enzyme activity, reshaping the gut microbiota community structure, and metabolic pathways. Phenylalanine was identified as a key metabolic regulatory factor. These findings not only expand the application paradigm of probiotics in the nutritional regulation of Lepidopteran insects but also provide new theoretical evidence for optimizing artificial feed-rearing systems.

## Supplementary Information

Below is the link to the electronic supplementary material.

Supplementary Material 1

## Data Availability

The raw sequence data of 16 S sequencing has been uploaded to NCBI Sequence Read Archive database under BioProject accession numbers PRJNA1248733 and PRJNA1247772. The raw data of metabolomics are available in the National Genomics Data Center (NGDC) OMIX repository under accession number OMIX009822.
