# Supplementary material for: Probiotic Bacillus subtilis enhances silkworm (Bombyx mori) growth performance and silk production via modulating gut microbiota and amino acid metabolism
Source: Anim Microbiome. 2025 Oct 3;7:103. doi: 10.1186/s42523-025-00473-1 (PMC12495873; doi:10.1186/s42523-025-00473-1)
Supplement: Supplementary file 1 — Supplementary Material 1 [file 42523_2025_473_MOESM1_ESM.docx]

*Supplementary data for*

Probiotic *Bacillus subtilis* Enhances Silkworm (*Bombyx mori*) Growth Performance and Silk Production via Modulating Gut Microbiota and Amino Acid Metabolism

**Pages: 5; Table: 1; Figures: 3**

Table S1. Alpha diversities of bacteria in the midgut of the silkworms

| Sample | Good’s coverage index | Shannon index | Simpson index | Chao1 index | Faith index | Observed species |
| --- | --- | --- | --- | --- | --- | --- |
| CK4I-1 | 0.99994 | 2.24890 | 0.46519 | 236.50 | 20.04 | 235.90 |
| CK4I-2 | 0.99995 | 2.00471 | 0.64285 | 104.32 | 29.26 | 103.80 |
| CK4I-3 | 0.99997 | 2.77502 | 0.77531 | 113.57 | 24.16 | 112.80 |
| BS4I-1 | 0.99997 | 1.53772 | 0.38483 | 118.65 | 15.34 | 118.40 |
| BS4I-2 | 0.99994 | 0.62179 | 0.12165 | 103.13 | 11.02 | 101.80 |
| BS4I-3 | 0.99994 | 0.49856 | 0.09176 | 117.78 | 20.12 | 116.00 |
| CK5I-1 | 0.99886 | 9.72701 | 0.99091 | 3309.72 | 257.44 | 3292.50 |
| CK5I-2 | 0.99880 | 9.47427 | 0.98903 | 3180.42 | 249.36 | 3160.20 |
| CK5I-3 | 0.99894 | 10.41930 | 0.99501 | 4064.45 | 311.47 | 4048.70 |
| BS5I-1 | 0.99953 | 10.23840 | 0.99552 | 2992.76 | 238.831 | 2980.90 |
| BS5I-2 | 0.99909 | 10.19440 | 0.99413 | 3748.80 | 290.36 | 3735.60 |
| BS5I-3 | 0.99957 | 10.02270 | 0.99433 | 3018.41 | 243.21 | 3011.30 |

CK-4I, CK group at 4 day of 4th instar; CK-5I, CK group at 3day of 5th instar; BS-4I, BS-2 group at 4 day of 4th instar; BS-5I, BS-2 group at 3day of 5th instar.


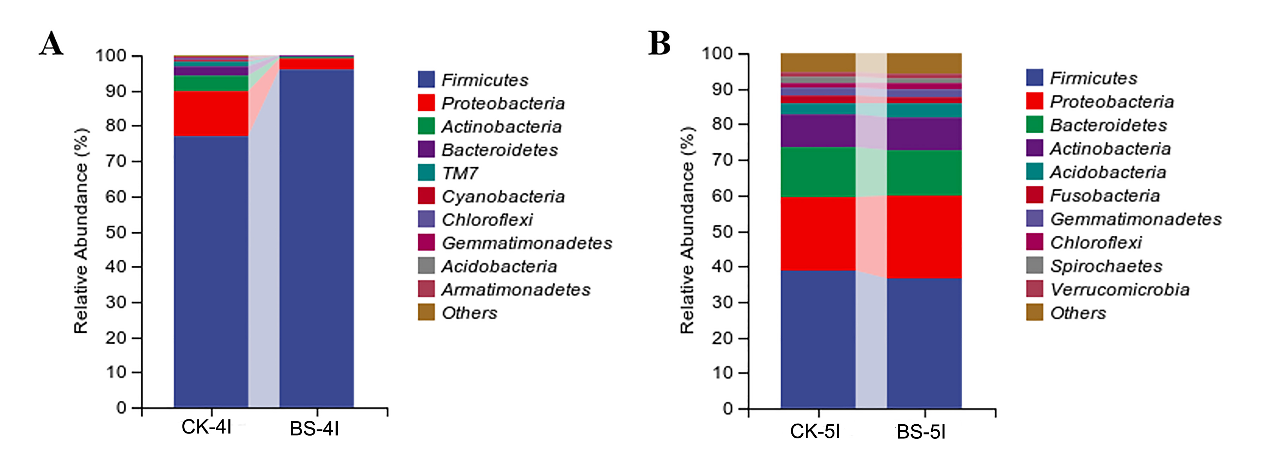


Fig. S1. The relative bacterial abundance at phyla-level in the midgut of the silkworms between the CK and BS-2 groups. CK-4I, CK group at 4 day of 4th instar; CK-5I, CK group at 3 day of 5th instar; BS-4I, BS-2 group at 3 day of 4th instar; BS-5I, BS-2 group at 3 day of 5th instar.


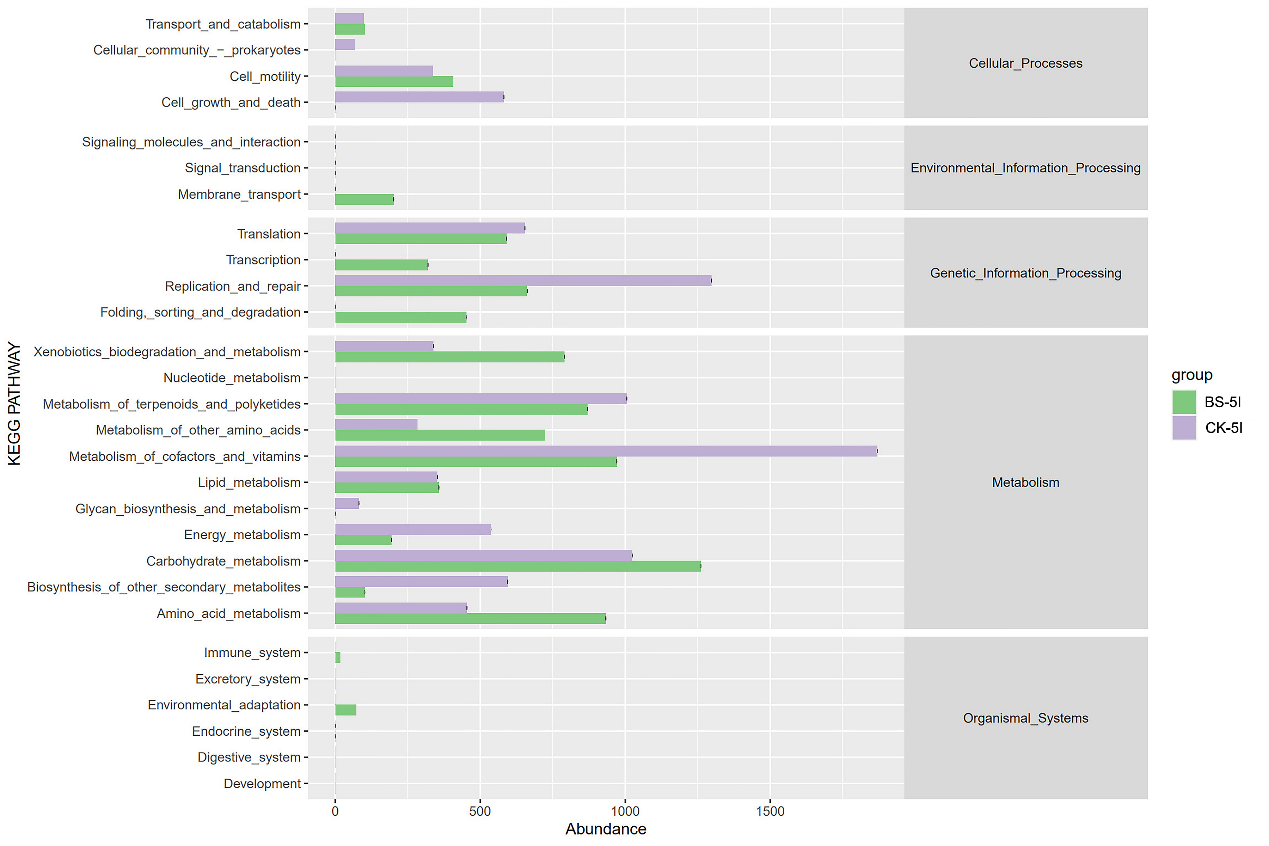


Fig. S2. KEGG analysis of gut bacterial metabolic pathways of the silkworms between the CK and BS-2 groups. CK-5I, CK group at 3 day of 5th instar; BS-5I, BS-2 group at 3 day of 5th instar.


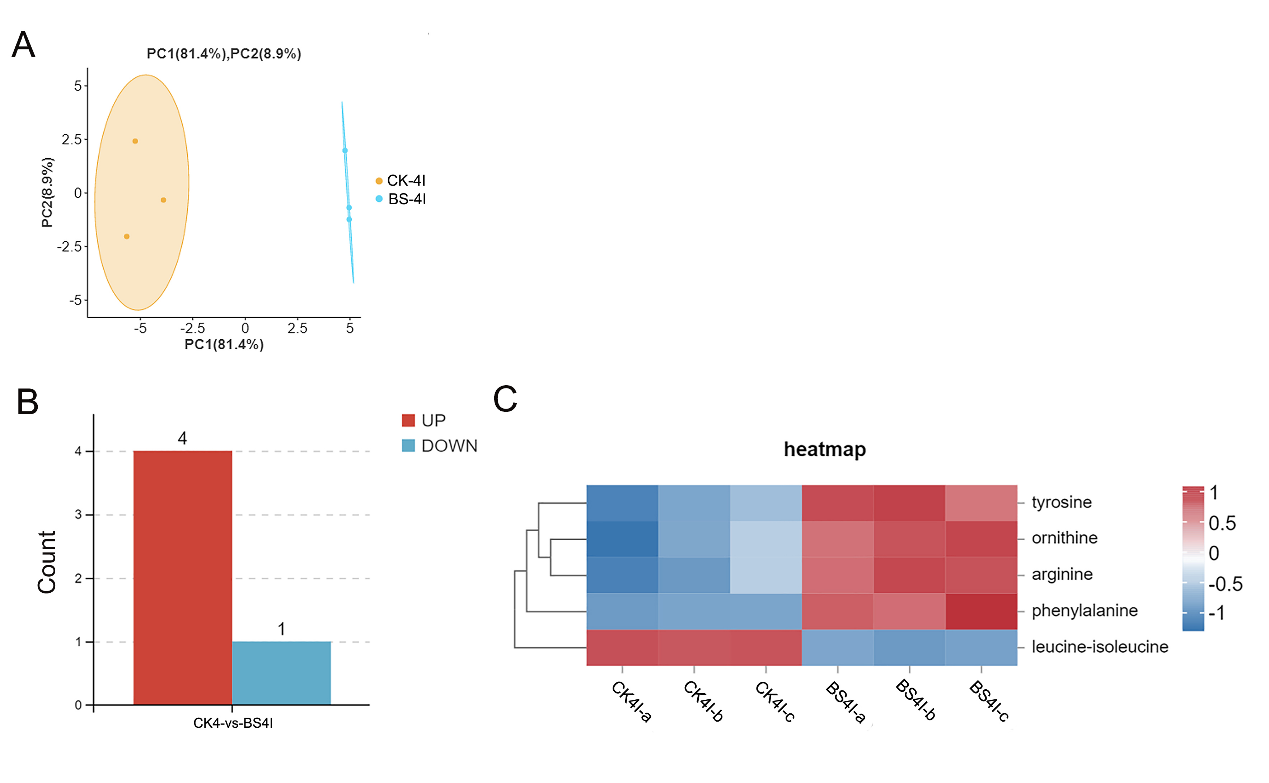


Fig. S3. Targeted metabolomics analysis of amino acids in the hemolymph of silkworm. (A) OPLS-DA analysis between CK group and BS-2 group. CK-4I, CK group at 4 day of 4th instar; BS-4I, BS-2 group at 3 day of 4th instar. (B) Differential amino acid counts between CK group and BS-2 group. (C) Heatmap of differential amino acid between CK group and BS-2 group.
